# Water Treadmill Training Ameliorates Neurite Outgrowth Inhibition Associated with NGR/RhoA/ROCK by Inhibiting Astrocyte Activation following Spinal Cord Injury

**DOI:** 10.1155/2022/1724362

**Published:** 2022-03-27

**Authors:** Xinwang Ying, Xiaolan Yu, Jintao Zhu, Xuqing Li, Yujun Zheng, Qingfeng Xie, Qiaoyun Wu, Shengcun Li, Jingjing Yue, Ye Zhou, Kecheng Zhou, Wenzhan Tu, Songhe Jiang

**Affiliations:** ^1^Department of Physical Medicine and Rehabilitation, The Second Affiliated Hospital and Yuying Children's Hospital of Wenzhou Medical University, Wenzhou, 325000 Zhejiang Province, China; ^2^Department of Intelligent Rehabilitation International (Cross-Strait) Alliance of Wenzhou Medical University, Wenzhou, 325000 Zhejiang Province, China; ^3^The First Affiliated Hospital of Zhejiang Chinese Medical University (Zhejiang Provincial Hospital of Traditional Chinese Medicine), China

## Abstract

Spinal cord injury (SCI) often results in damage to or degeneration of axons. Crosstalk between astrocytes and neurons plays a pivotal role in neurite outgrowth following SCI. Rehabilitative training is a recognized method for the treatment of SCI, but the specific mechanism underlying its effect on axonal outgrowth in the central nervous system (CNS) has not yet been determined. A total of 190 adult male SD rats weighing 200–250 g were randomly divided into eight groups for use as animal models of SCI. Rats were subjected to water treadmill training (TT) for 7 or 14 d. The Basso-Beattie-Bresnahan (BBB) motor function scale, hematoxylin-eosin (HE) staining, Nissl staining, Western blotting, and immunofluorescence were used to measure tissue morphology and the degree of neurological deficit and to determine quantitative expression and accurate localization of the corresponding proteins. We found that TT decreased tissue structure damage and improved functional recovery. TT also promoted the regeneration of neurons and reduced SCI-induced apoptosis SCI around the lesion, as well as significantly increasing the expression of GAP43 and NF200 after SCI. In addition, TT significantly inhibited the injury-induced increase in the expression of proinflammatory factors. Moreover, TT reduced the activation of astrocytes and microglia, accompanied by the reduced expression of C3d and increased expression of S100A10. Finally, TT effectively reduced the level of chondroitin sulfate proteoglycan (CSPG) surrounding the lesion and inhibited the NGR/RhoA/ROCK signaling pathway in neurons after SCI. Overall, we found that TT played a novel role in recovery from SCI by promoting axonal outgrowth associated with NGR/RhoA/ROCK signaling by inhibiting astrocyte activation after SCI.

## 1. Introduction

Spinal cord injury (SCI) often results in damage to or degeneration of axons. The regenerative ability of damaged axons is poor, and regenerated axons are not well guided to form functional connections [1]. Therefore, it is important to find a treatment that promotes axon regeneration and provides corresponding guidance signals to regenerated axons so that they can reform an effective conduction circuit, thus promoting functional recovery during rehabilitation after SCI.

In the ischemic and inflammatory microenvironment caused by SCI, the phenotype of naive astrocytes (NAs) changes; the cells first become reactive astrocytes (RAs) and then turn into scar-formed astrocytes (SAs) [2, 3]. RAs can be divided into toxic A1 astrocytes, which induce rapid death in neurons and oligodendrocytes, and neuroprotective A2 astrocytes, which promote neuronal survival and tissue repair [4]. RAs proliferate, migrate, and transform into SAs that form astrocytic scars around the lesion epicenter [5, 6]. Glial scarring and chondroitin sulfate proteoglycan (CSPG), one of the hallmark genes of RAs, are the core barriers against axonal regeneration in damaged neurons caused by SCI [7, 8]. As such, elucidating the role of crosstalk between astrocytes and neurons after SCI may be helpful in developing new therapeutic strategies.

Nogo receptor 1 (NGR1) and NGR3 are two receptors of myelin-related inhibitors [9]. When these receptors interact with CSPG, they activate the Rho/ROCK signaling pathway and induce the collapse of axonal growth cones [10]. Rho family proteins are a group of guanosine triphosphate-binding proteins with relative molecular weights of approximately 20-30 kDa. The main function of Rho proteins is to regulate recombination of the actin cytoskeleton through the downstream effector Rho kinase (ROCK) [11, 12]. Almost no protein expression of RhoA/ROCK in mature neurons has been reported, but the expression of RhoA increases significantly (>10 times) after SCI [13]. RhoA begins to appear as early as 1.5 h after injury and continues to be highly expressed for 4 weeks [9]. Tomas et al. [14] and Sabine et al. [15] have conducted in vivo SCI experiments and reached a similar conclusion: the processes of nerve cells in the injured area contain have high levels of activated RhoA and ROCK expression, and blocking these factors can promote axonal growth. In addition, the in vitro use of specific ROCK inhibitors indicated that ROCK has a negative regulatory effect on the activity of growth cones [16]. Together, these studies show that the RhoA/ROCK pathway in neurons plays an indispensable role in the inhibition of axonal regeneration.

Rehabilitative training is currently one of the most effective nondrug treatments for SCI, but the mechanism has not been elucidated. Studies have confirmed that rehabilitative training can increase the expression of neurotrophic factors in the central nervous system (CNS), which is beneficial for corresponding nerve growth [17–19]. Surprisingly, Irin et al. reported that compulsory exercise therapy also promotes the sprouting of axonal lateral branches [20]. It has also been reported that rehabilitative training can promote axonal regeneration in peripheral nerve injury [21], but the specific mechanism by which rehabilitative training affects axonal outgrowth in the CNS has not been determined. In this study, we combined a treadmill with swimming (to avoid potential injuries caused by early intensive training) and used the water treadmill [22] to explore the mechanism underlying neurite outgrowth after SCI.

## 2. Materials and Methods

### 2.1. Antibodies

5-Bromo-20-deoxyuridine (BrdU), glial fibrillary acidic protein (GFAP), ionized calcium binding adapter molecule 1 (Iba1), and neurofilament-200(NF200) antibodies were purchased from Abcam (MC, UK). Ras homolog gene family, member A (RhoA), anti-C3d, anti-NGR, interleukin-1*β* (IL-1*β*), interleukin-6 (IL-6), tumor necrosis factor *α* (TNF*α*), growth associated protein-43 (GAP43), and anti-GAPDH antibodies were purchased from Affinity (OH, USA). S100 calcium-binding protein A10 (S100A10) and anti-ROCK2 were purchased from ABclonal Technology Co., Ltd. (Wuhan, China). CSPGs was purchased from Merck Chemicals (Shanghai, China) Co., Ltd. Neuron-specific nuclear protein (NeuN) was purchased from Novus (Co, USA). Antitubulin was purchased from Proteintech (IL, USA). Y-27632 dihydrochloride was purchased from APExBIO (Tx, USA).

### 2.2. Animals

A total of 190 adult male SD rats weighing 200–250 g were obtained from Shanghai Laboratory Animal Centre. Rats were randomly divided into eight groups: sham-operated (*n* = 40; group S), S+ Y-27632 (*n* = 10; group SI), S + TT (*n* = 10; group ST), 7 d after SCI (*n* = 30; group M7), M+ Y-27632 (*n* = 10; group MI), 14 d after SCI (*n* = 30; group M14), SCI + TT for 7 d (*n* = 30; group TM7), and SCI + TT for 14 d (*n* = 30; group TM14). Rats are kept in a controlled environment and fed food and water regularly. All protocols were approved by the Animal Research Committee of Wenzhou Medical University.

### 2.3. SCI Model

Rats were kept anesthetized using 2% pentobarbital sodium (30 mg/kg). Then, the T9-T11 area on the back of the rat was quickly scraped off, an incision of about 1 cm was made with a scalpel, and the lamina was peeled off with rongeurs to expose the corresponding spinal cord. A New York University Impactor (10 g × 20 cm) was used to impact the exposed site except for group S. Spinal cord bruising, lower limb trembling, and tail wagging indicate the success of the model. Finally, the wound is quickly sutured with absorbable surgical thread. In the following experimental phase, the bladder was emptied, and the urethra was cleaned every morning and evening.

### 2.4. Water Treadmill Training and Inhibitor Injection

The rats will have 3 d of adaptive treadmill training before the establishment of the model. The setting of the treadmill (Wenzhou Xinglong Stainless Steel Co, Ltd., Zhejiang, China): 10-15 m/min, 5 min/round, 3 rounds in total, 5 min interval between rounds, AND water temperature is maintained at 30°C. The following day after SCI, the rats in the ST and TM groups were treated with TT for 7 d or 14 d, respectively. Each time before experiment, rats were put into water at 30°C for defecation. After the experiment, the rats will be cleaned and dried and then put back into the cage. It is reported that Y-27632, an inhibitor, can effectively inhibit RhoA/ROCK signaling pathway and promote axonal growth [23]. In our study, the SI and IM groups were injected with Y-27632 (0.1 mg/kg) every 24 hours after SCI, and the other groups were injected with the same amount of normal saline.

### 2.5. Behavioral Tests

Motor function was conducted by two independent examiners who were blinded to the treatment groups using the Basso-Beattie-Bresnahan (BBB) open-field test, which ranges from 0 (complete paralysis) to 21 (normal locomotion). The animals in each group (*n* = 10) were tested at 1, 3, 7, and 14 d following injury.

### 2.6. Tissue Preparation

Rats from each group were sacrificed at 7 and 14 d after SCI. After 2% pentobarbital sodium deep anesthesia, rats were transcardially perfused with 0.9% sodium chloride at 4°C, followed by 4% paraformaldehyde in 0.1 M phosphate buffer (PB, pH 7.4). For the rats to be stained with Brdu, 1% solution (10 mg/ml) was injected intraperitoneally 3 times every 4 h before the rats were sacrificed. Then, the T9-T11 spinal cord tissue was removed from the rats quickly, stored in the same fixative for 24 hours (4°C), and immersed in 0.1 M phosphate-buffered 20% and 30% sucrose overnight at 4°C, respectively. Successive sections (15 *μ*m thick) were frozen and stored for subsequent experiments.

### 2.7. Hematoxylin-Eosin (HE) and Nissl Staining

Prepared sections (*n* = 5 per group) were naturally air-dried for 3 min. Then, sections were stained with hematoxylin and eosin for HE staining and cresyl violet for Nissl staining, respectively. Finally, the Olympus BH-2 microscope (Olympus Optics, London, UK) was used to capture the image and the size of cavity area was quantitatively counted.

### 2.8. TUNEL Staining

Prepared sections (*n* = 5 per group) were collected for TUNEL staining. Permeabilized the slides for 10 min at room temperature and washed the slides three times for 15 min. Then, an In Situ Cell Death Detection Kit (Roche Molecular Biochemicals) was used to detect the apoptotic cells, using a microscope (BX51, Olympus) to observe the images. The apoptosis positive cells were counted quantitatively using Image-Pro Plus 6.0 analysis software.

### 2.9. RT-PCR

The primer sequences were 5′TGAGGAGAAGAAGGGCGAAGGG 3′ (forward), 5′AGGACGGCGAGTTATCAGTGGTAG3′ (reverse) for GAP43 and 5′ACGCTGCAGTCAGAGGAGTG3′ (forward), 5′CTGCCGCCGGTACTCAGTTA 3′ (reverse) for NF200. High Capacity RNA-to-cDNA Master Mix (A3500, Promega) was applied to reverse transcribed the one microgram of total RNA. Real-time amplification was performed using SYBR Green (QPK-212, Tokyo, Japan) and a Light Cycler480 (Roche, USA). To amplify genomic DNA, PCR was performed under the following conditions: reverse transcription at 50°C for 3 min, DNA polymerase activation, and RT enzyme inactivation at 95°C for 5 min, followed by 45 cycles of denaturation at 95°C for 10 s, primer annealing at 60°C for 10 s, and elongation at 72°C for 10 s. Comparative mRNA expression levels were expressed as 2^-*ΔΔ*Ct^.

### 2.10. Western Blot Analysis

At 7 and 14 d after injury, rats (*n* = 5 each group) were sacrificed, and a 5 mm length of the T10 spinal cord was obtained. After the tissue is fully homogenized, the supernatant is taken. Then, the extracted protein concentration was quantified by the bicinchoninic acid (BCA) assay kit. A separation gel suitable for the molecular weight of the protein was prepared for electrophoresis, and separated proteins were transferred to a PVDF membrane (Bio-Rad, Hercules, CA, USA). After a 90-minute 5% milk closure, the membrane was washed three times with Tris-buffered saline solution with Tween (TBST)and incubated with the corresponding primary antibodies at 4°C for 16-24 h: GAP43 (1 : 1000), NF200 (1 : 1000), IL-1*β* (1 : 500), IL-6 (1 : 1500), TNF*α* (1 : 500), GFAP (1 : 1000), Iba1 (1 : 1000), C3d (1 : 500), S100A10 (1 : 500), CSPGs (1 : 500), NGR (1 : 500), RhoA (1 : 1000), ROCK2 (1 : 500), tubulin (1 : 1000), and GAPDH (1 : 1000). The secondary antibodies were incubated for 2 h and washed with TBST 3 times. All experiments were repeated three times. Images were visualized by the ChemiDicTM XRS + Imaging System (Bio-Rad).

### 2.11. Immunofluorescence Staining

Prepared sections were naturally air-dried and washed three times in 0.01 M PBS, blocked with 10% normal goat serum at room temperature for 1 h, and incubated with rabbit anti-GAP43 (1 : 200), mouse anti-NF200 (1 : 200), mouse anti-Brdu (1 : 200), goat (rabbit) anti-NeuN (1 : 100), mouse (rabbit) anti-GFAP (1 : 500), rabbit anti-Iba1(1 : 300), rabbit anti-C3d (1 : 200), rabbit anti-S100A10 (1 : 20), mouse anti-CSPGs (1 : 200), rabbit anti-ROCK2(1 : 100), rabbit anti-RhoA (1 : 200), and rabbit anti-NGR (1 : 200) overnight at 4°C. The second day, the sections were incubated with secondary antibodies: cy3-conjugated goat anti-rabbit (1 : 300), Alexa Flour 488-conjugated goat anti-mouse (1 : 400), cy3-conjugated goat anti-mouse (1 : 300), and Alexa Flour 488-conjugated goat anti-rabbit (1 : 400) at room temperature for 50 min. Nuclei were counterstained with 4,6-diamidino-2-phenylindole (DAPI). The fluorescence signal was observed under laser confocal microscopy. Five fields on each of three slides per animal were randomly selected for visualization and analysis performed using ImageJ software (National Institutes of Health, Bethesda, MD, USA).

### 2.12. Statistical Analysis

All experimental values are presented as the mean ± SD. The Kolmogorov-Smirnov test was used as a normality test, with *p* > 0.05 indicating a normal distribution. Levene's test was used as a test of homogeneity of variance, with *p* > 0.05 used to indicate homogeneous variance, and vice versa. When there were two experimental groups, Student's *t*-test was used. Statistical differences among groups were analyzed using two-way ANOVA followed by Tukey's test. BBB scores were detected using repeated measurement two-way ANOVA with group and time as factors, followed by Tukey's test to detect differences between groups. SPSS 16 statistical software was used for statistical analysis, and *p* < 0.05 was considered statistically significant.

## 3. Results

### 3.1. TT Decreased Tissue Structure Damage and Improved Functional Recovery after SCI

**Figure 1:**
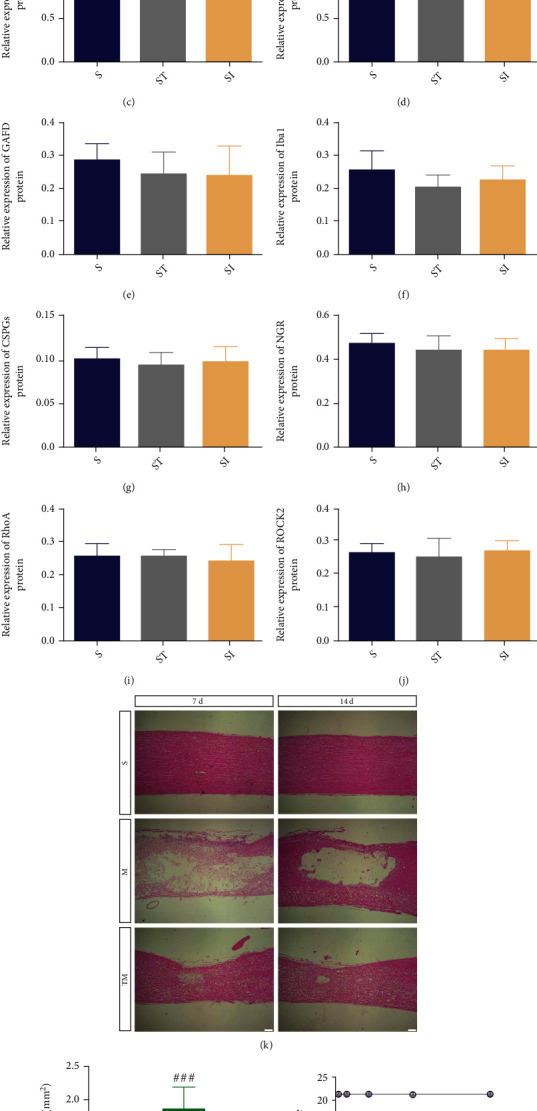
TT decreased tissue structure damage and improved functional recovery after SCI. (a)–(j) Representative Western blots and quantification data for NF200/tubulin, GAP43/tubulin, GFAP/tubulin, Iba1/tubulin, CSPGs/GAPDH, NGR/GAPDH, RhoA/GAPDH, and ROCK2/CREB in each group. *Columns* represent the mean ± SD, *n* = 5. (k) HE-stained longitudinal sections of the spinal cord at 7 d and 14 d after SCI. Scale bar = 200 *μ*m. (l) Quantification of the cavity area; *columns* represent the mean ± SD (*n* = 5). (m) BBB scores in the S, SI, ST, M, and TM groups. ^#^*p* < 0.05 for the M group versus the S group, ^∗^*p* < 0.05 for the TM group versus the M group.

We investigated whether TT and the ROCK inhibitor Y27632 affected neural function in the S group. Compared to those in the S group, the expression of NF200, GAP43, GFAP, Iba1, CSPGs, NGR, RhoA, and ROCK2 in both the SI and ST groups was not significantly different (S vs. SI; S vs. ST: *p* > 0.05) (Figures 1(a)–1(j)). At 7 and 14 d after injury, HE staining was used to observe the morphological and histological differences in the T9-T11 spinal cord segment (Figure 1(k)). The tissue in the S group was closely packed, but the structural integrity of tissue in the M group was clearly destroyed, with the injured tissue forming pathological holes after digestion and absorption. Quantitative analysis at 14 d showed that the cavity area in the TM group was significantly smaller than that in the M group after TT (Figure 1(l)) (TM vs. M: *p* < 0.001). Functional recovery was evaluated by BBB motor functional scoring at 1, 3, 7, and 14 d after SCI. The results (Figure 1(m)) showed that the BBB scores in the TM group were significantly higher than those in the M group at 7 and 14 d after SCI (TM vs. M: 7 d, *p* < 0.01; 14 d, *p* < 0.001), and that the BBB scores in the SI and ST groups did not significantly differ from that of the S group (SI and ST vs. S: *p* > 0.05). These results indicate that TT can significantly improve functional recovery and preserve tissue.

### 3.2. TT Promoted the Regeneration of Neurons and Reduced Apoptosis around the Lesion

**Figure 2:**
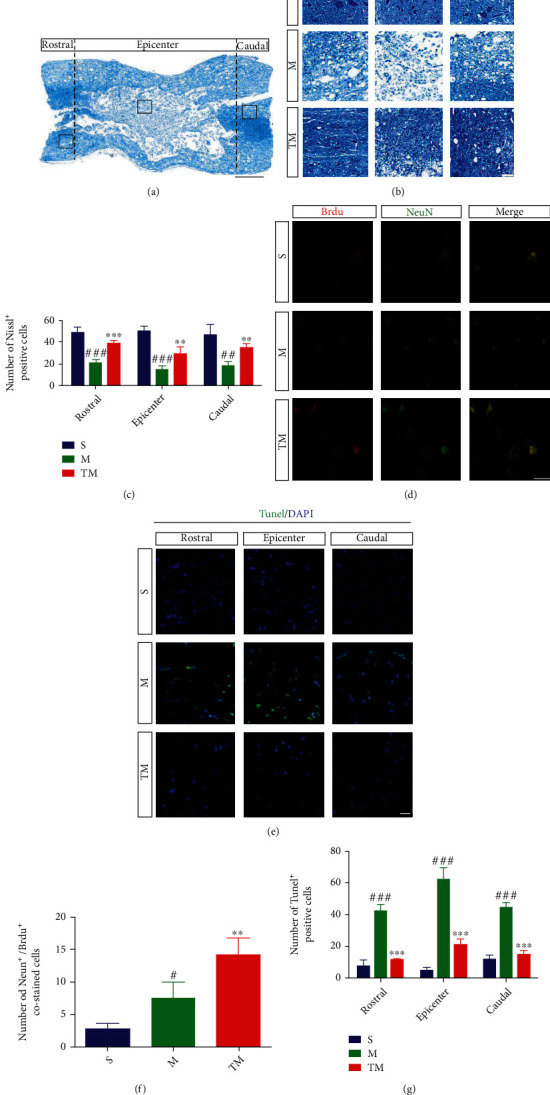
TT promoted the regeneration of neurons and reduced apoptosis around the lesion. (a) Schematic diagram of the regions sampled after SCI. Scale bar = 500 *μ*m. (b) Nissl staining in the S, M, and TM groups from different regions at 7 d. Scale bar = 50 *μ*m. (c) Quantification of the Nissl staining-positive cells; *columns* represent the mean ± SD (*n* = 5). (d) Double staining at 14 d after SCI for BrdU/NeuN. Red: BrdU; green: NeuN. Scale bar, 20 *μ*m. (e) TUNEL staining in the rostral/epicenter/caudal regions at 7 d after SCI. The scale bar represents 20 *μ*m. (f, g) Quantification data of BrdU^+/^NeuN^+^ and TUNEL^+^ cells in each group*. Columns* represent the mean ± SD, *n* = 5.

To observe neuronal damage, rostral to caudal Nissl staining was performed at 7 d in the different groups (Figures 2(a) and 2(b)). As shown in Figure 2(c), the number of Nissl staining-positive cells were significantly lower in the rostral, epicenter, and caudal areas in the M group than in the S group (M vs. S: rostral, *p* < 0.001; epicenter, *p* < 0.001; caudal, *p* < 0.01). However, the number of Nissl staining-positive cells was significantly higher in these areas in the TM group than the M group (TM vs. M: rostral, *p* < 0.001; epicenter, *p* < 0.01; caudal, *p* < 0.01). We further measured apoptosis (TUNEL staining) in each group 7 d after SCI (Figure 2(e)). The number of apoptotic cells was significantly higher after SCI in the S group than the M group (M vs. S: rostral/epicenter/caudal, *p* < 0.001) but was significantly lower in the TM group than the M group (TM vs. M: rostral/epicenter/caudal, *p* < 0.001) (Figure 2(g)). At 14 d after injury, we used the neuronal marker NeuN and the regeneration marker BrdU to observe neuronal regeneration (Figure 2(d)). As shown in Figure 2(f), nerve regeneration (NeuN^+^/BrdU^+^ colabeled cells) in the TM group was increased compared to that in the M group (TM vs. M: *p* < 0.01).

### 3.3. TT Promoted Axonal Outgrowth after SCI

**Figure 3:**
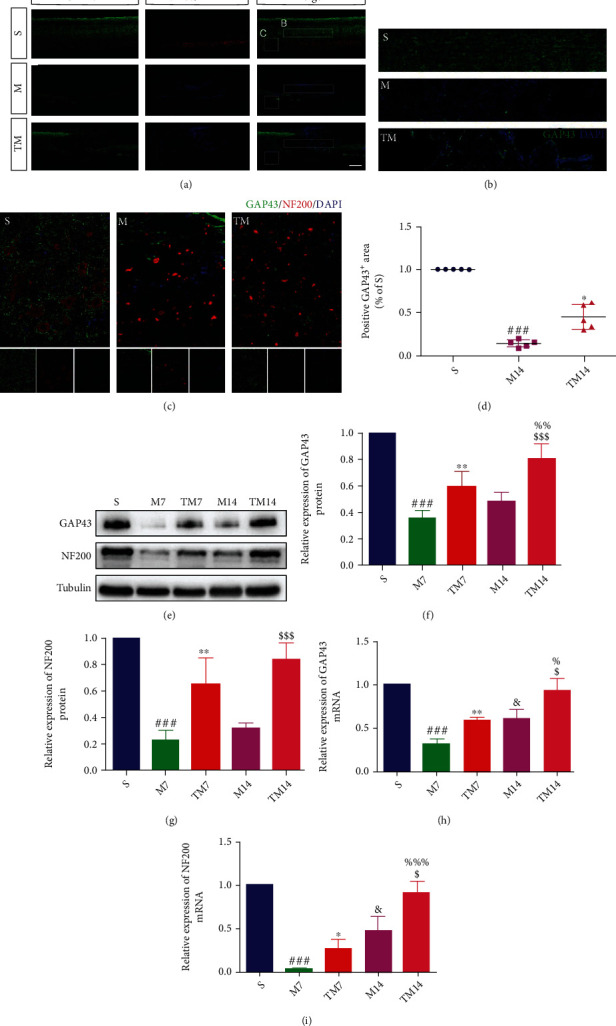
TT promoted axonal outgrowth after SCI. (a)–(c) Double staining of spinal cord sections in each group of rats for GAP43 (green)/NF200 (red)/DAPI (blue). Scale bars are 500 *μ*m (a, b) and 50 *μ*m (c). (d) Quantification of GAP43^+^ cells; *columns* represent the mean ± SD (*n* = 5). (e) Western blots and (f, g) quantification of GAP43 and NF200 proteins in each group; *columns* represent the mean ± SD (*n* = 5). (h, i) Representative RT–qPCR analysis of GAP43 and NF200 mRNA expression at 7 d and 14 d in the different groups; *columns* represent the mean ± SD, *n* = 5. ^&^*p* < 0.05 for the M14 group versus the M7 group, ^$^*p* < 0.05 for the TM14 group versus the M14 group, ^%^*p* < 0.05 for the TM14 group versus the TM7 group.

GAP43/NF200 staining was used to determine the effects of TT on axonal outgrowth around the epicenter at 14 d after SCI (Figures 3(a)–3(c)). In the penumbra (1-3 mm adjacent to the epicenter), we found that the area of GAP43-positive cells decreased significantly after SCI (Figure 3(d)); in contrast, there was higher GAP43 expression levels in the TM group than in the M group (M vs. S: *p* < 0.01; TM vs. M: *p* < 0.05). Moreover, Western blotting was also performed to measure the protein expression of GAP43 and NF200 in each group (Figure 3(e)). The results (Figures 3(f) and 3(g)) showed that the GAP43 and NF200 protein expression in the M group was decreased after SCI at 7 d and 14 d compared to the expression levels in the S group, and that the expression levels in the TT group were between those of the other two groups (GAP43, M_7_ vs. S: *p* < 0.001, TM_7_ vs. M_7_: *p* < 0.01, TM_14_ vs. M_14_: *p* < 0.001; NF200, M_7_ vs. S: *p* < 0.001, TM_7_ vs. M_7_: *p* < 0.001, TM_14_ vs. M_14_: *p* < 0.001). RT–qPCR analysis (Figures 3(h) and 3(i)) found significant differences in GAP43 and NF200 mRNA expression levels between the M and TM groups (GAP43 mRNA, M_7_ vs. S: *p* < 0.001, TM_7_ vs. M_7_: *p* < 0.01, TM_14_ vs. M_14_: *p* < 0.05; NF200 mRNA, M_7_ vs. S: *p* < 0.001, TM_7_ vs. M_7_: *p* < 0.05, TM_14_ vs. M_14_: *p* < 0.05).

### 3.4. TT Inhibited the Inflammatory Activation of Microglia and Astrocytes

**Figure 4:**
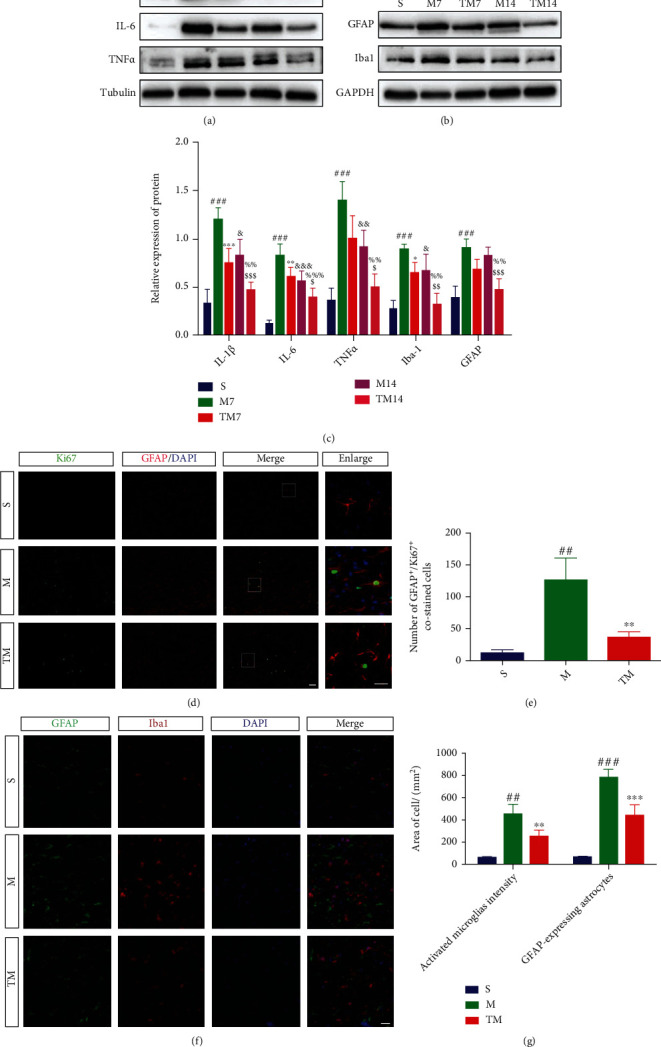
TT inhibited the inflammatory activation of microglia and astrocytes. (a, b) Western blots and (c) quantification of IL-4, IL-6, TNF*α*, GFAP, and Iba1 proteins in each group after SCI; *columns* represent the mean ± SD (*n* = 5). (d) Double staining (green: Ki67; red: GFAP; blue: DAPI) and (e) quantification of GFAP^+^/Ki67^+^ cells; *columns* represent the mean ± SD (*n* = 5). (f) Double staining (green: GFAP; red: Iba1; blue: DAPI) and (g) quantification of the positive cell area.

In the T9-T11 spinal cord segment, the total protein expression of proinflammatory factors (IL-1*β*, IL-6, and TNF-*α*) and glial cells (GFAP and Iba1) was measured by Western blotting (Figures 4(a) and 4(b)). We normalized the protein levels and then analyzed the data in comparison with the corresponding group (IL-1*β*, M_7_ vs. S: *p* < 0.001, TM_7_ vs. M_7_: *p* < 0.001, TM_14_ vs. M_14_: *p* < 0.001; IL-6, M_7_ vs. S: *p* < 0.001, TM_7_ vs. M_7_: *p* < 0.01, TM_14_ vs. M_14_: *p* < 0.05; TNF-*α*, M_7_ vs. S: *p* < 0.001, TM_7_ vs. M_7_: *p* < 0.05, TM_14_ vs. M_14_: *p* < 0.05; GFAP, M_7_ vs. S: *p* < 0.001, TM_7_ vs. M_7_: *p* < 0.01, TM_14_ vs. M_14_: *p* < 0.001; Iba1, M_7_ vs. S: *p* < 0.001, TM_7_ vs. M_7_: *p* < 0.05, TM_14_ vs. M_14_: *p* < 0.01) (Figure 4(c)). To examine astrocyte proliferation, we used Ki67 to fluorescently label proliferating cells and GFAP to fluorescently label astrocytes and then analyzed the degree of costaining (Figure 4(d)). The number of GFAP^+^/Ki67^+^ cells significantly increased at 7 d after injury, but this outcome was improved by TT (M vs. S: *p* < 0.01; TM vs. M: *p* < 0.01) (Figure 4(e)). In addition, we also performed GFAP/Iba1 costaining experiments to observe the activation and positional connection of glial cells (Figure 4(f)). As shown in Figure 4(g), astrocytes and microglia were activated after SCI, but TT significantly decreased the number of activated glial cells (GFAP-expressing astrocytes, M vs. S: *p* < 0.001, TM vs. M: *p* < 0.001; activated microglial microglia intensity, M vs. S: *p* < 0.01, TM vs. M: *p* < 0.01).

### 3.5. TT Inhibited A1-Type Reactive Astrocytes and Promoted A2-Type Reactive Astrocytes around the Lesion

**Figure 5:**
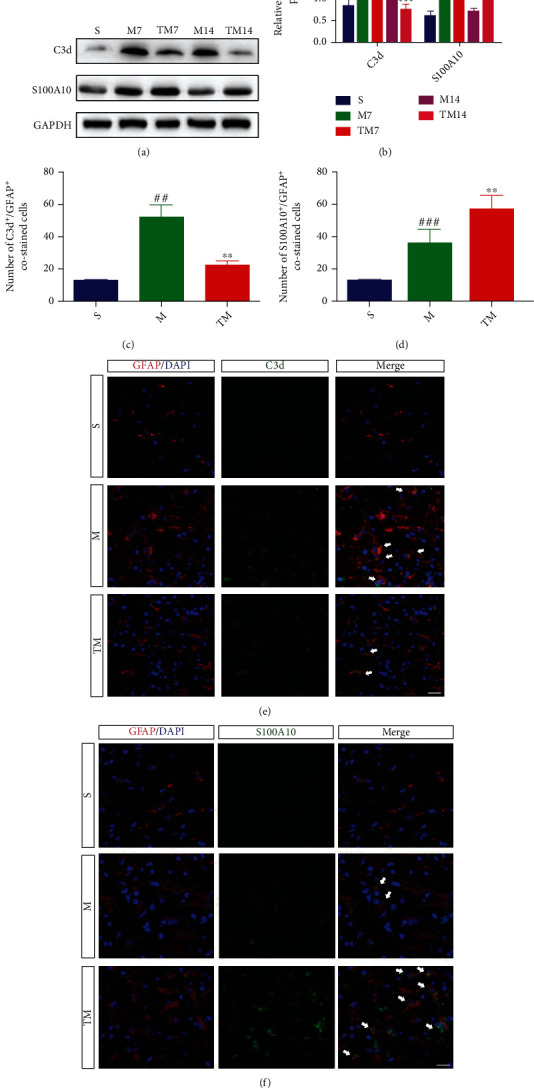
TT inhibited A1-type reactive astrocytes and promoted A2-type reactive astrocytes around the lesion. (a) Western blots and (b) quantification of C3d and S100A10 proteins in each group after SCI; *columns* represent the mean ± SD (*n* = 5). (c, d) Quantification of C3d^+^; S100A10^+^/GFAP^+^ cells. (e, f) Double staining of sections in each group for C3d; S100A10 (green)/GFAP (red)/DAPI (blue). Scale bars are 200 *μ*m. White arrowheads indicate costaining of C3d/S100A10 and GFAP.

In the penumbra, the protein expression levels of the A1-type astrocyte marker C3d and the A2-type astrocyte marker S100A10 were measured by Western blotting (Figure 5(a)). The protein expression of C3d was markedly higher in the M group than in the S group (M vs. S: *p* < 0.001), and this effect was reduced by TT (TM vs. M: 7 d, *p* < 0.01; 14 d, *p* < 0.001). Additionally, the S100A10 protein expression level was higher in the M group than the S group (M vs. S: *p* < 0.01) and higher in the TM group than the M group (TM vs. M: 7 d, *p* < 0.001; 14 d, *p* < 0.05) (Figure 5(b)). We further performed costaining of GFAP/C3d and GFAP/S100A10 (Figures 5(e) and 5(f)). As shown in Figure 5(c), the C3d expression in astrocytes increased significantly after injury but decreased in response to TT (M vs. S: *p* < 0.01; TM vs. M: *p* < 0.01). In contrast, the protein expression of S100A10 in astrocytes increased after SCI and further increased after TT intervention (M vs. S: *p* < 0.001; TM vs. M: *p* < 0.01) (Figure 5(d)). These results suggest that TT can inhibit A1 astrocyte activation to a certain extent and can promote A2 astrocyte activation.

### 3.6. TT Inhibited the Expression of RhoA/ROCK Induced by Astrocyte Activation

**Figure 6:**
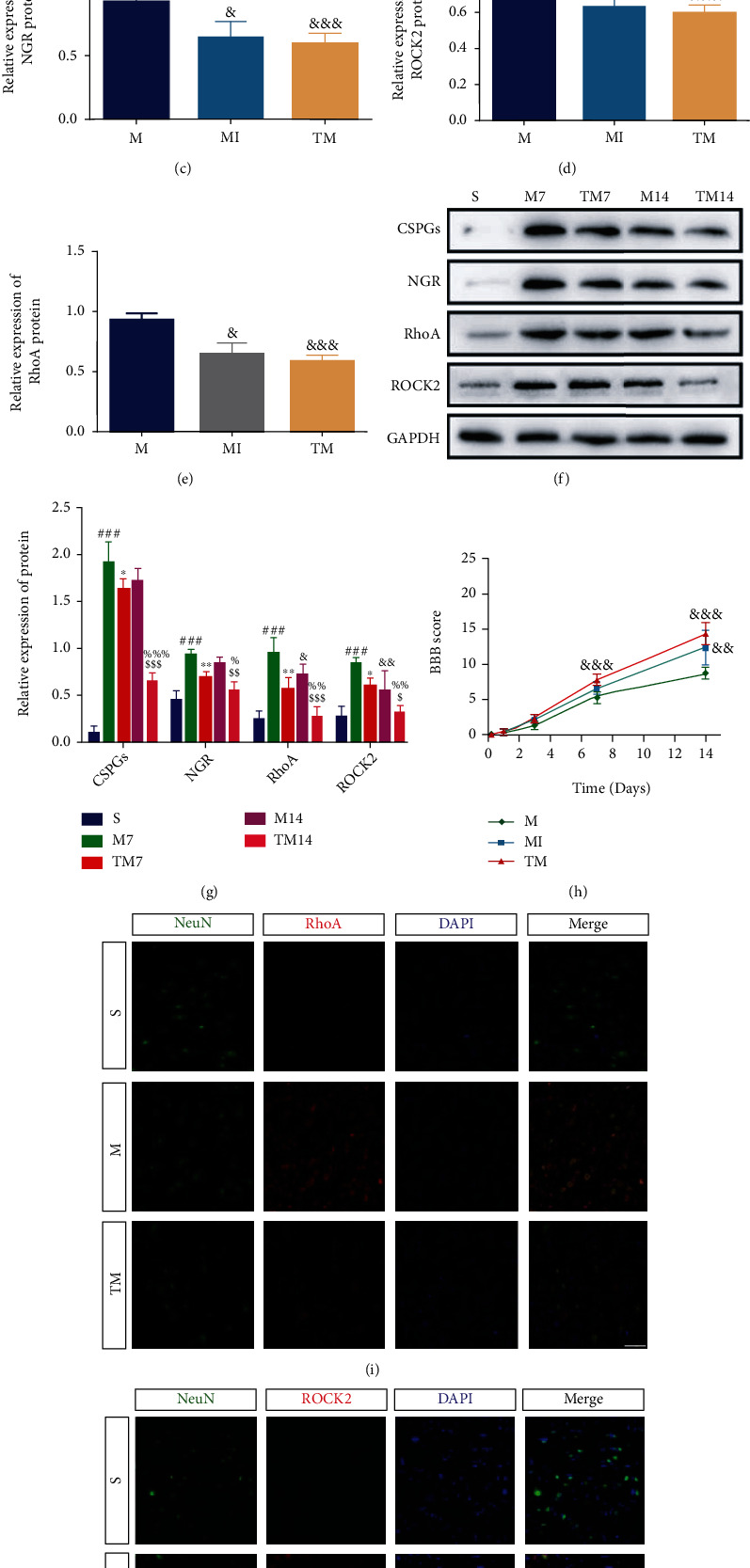
TT inhibited the expression of RhoA/Rock induced by astrocyte activation. (a)–(e) Representative Western blots and quantification data for CSPGs/GAPDH, NGR/GAPDH, RhoA/GAPDH, and ROCK2/CREB in each group*. Columns* represent the mean ± SD, *n* = 5. (f) Western blots and (g) quantification of CSPG, NGR, RhoA, and ROCK2 proteins in each group after SCI; *columns* represent the mean ± SD (*n* = 5). (h) BBB scores in M, MI, and TM groups. (i, j) Double staining at 14 d after SCI for RhoA; ROCK2//NeuN/DAPI. Red: RhoA/ROCK2; green: NeuN; blue: DAPI. Scale bars are 50 *μ*m.

**Figure 7:**
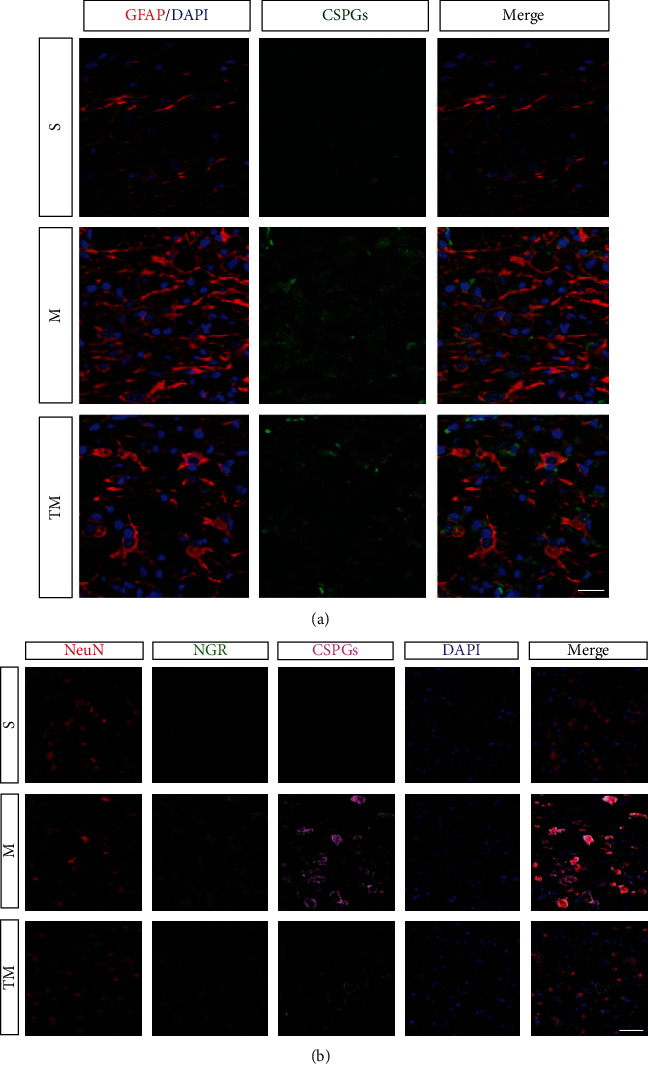
(a) Double staining for GFAP/CSPGs/DAPI. Red: GFAP; green: CSPGs; blue: DAPI. Scale bar = 20 *μ*m. (b) Localization and expression of NeuN/NGR/CSPGs/DAPI. red: NeuN; green: NGR; pink: CSPGs; blue: DAPI. White arrowheads indicate costaining of NeuN/NGR/CSPGs/DAPI. Scale bar, 50 *μ*m.

To compare the effects of the inhibitor and TT on neurological function after SCI, BBB scores and NGR/RhoA/ROCK signaling pathway molecules were examined (Figure 6(a)). The results (Figure 6(b)–6(e)) suggest that both signaling pathway molecules and neurobehavioral scores are improved after the use of the inhibitor and of TT, but the effect of TT is more significant (CSPGs, MI vs. M: *p* < 0.05, TM vs. M: *p* < 0.01; NGR, MI vs. M: *p* < 0.05, TM vs. M: *p* < 0.001; RhoA, MI vs. M: *p* < 0.001, TM vs. M: *p* < 0.001; ROCK2, MI vs. M: *p* < 0.05, TM vs. M: *p* < 0.001). To study the crosstalk between astrocytes and neurons, CSPG and NGR/RhoA/ROCK signaling pathway protein expression in the penumbra was measured by Western blotting at 7 and 14 d (Figure 6(f)). As shown in Figure 6(g), after injury, the expression of CSPG increased significantly, but the expression of CSPG in the TT group was significantly lower than that in the M group; the effect of TT became more obvious at 14 d (M_7_ vs. S: *p* < 0.001, TM_7_ vs. M_7_: *p* < 0.05, TM_14_ vs. M_14_: *p* < 0.001). We found that the expression of NGR/RhoA/ROCK signaling pathway molecules was strikingly similar to the trend in the CSPG expression (NGR, M_7_ vs. S: *p* < 0.001, TM_7_ vs. M_7_: *p* < 0.01, TM_14_ vs. M_14_: *p* < 0.01; RhoA, M_7_ vs. S: *p* < 0.001, TM_7_ vs. M_7_: *p* < 0.01, TM_14_ vs. M_14_: *p* < 0.001; ROCK2, M_7_ vs. S: *p* < 0.001, TM_7_ vs. M_7_: *p* < 0.05, TM_14_ vs. M_14_: *p* < 0.05). To further determine the localized expression of CSPG, we costained the tissue for GFAP and CSPG (Figure 7(a)). Consistent with the previous trend in the protein expression, astrocytes produced CSPG around the epicenter after SCI, and this effect was significantly improved by TT. We also carried out localized multilabel staining of NGR/RhoA/ROCK signaling pathway factors at 14 d to further verify the experimental results. As shown in Figures 6(i) and 6(j), the expression of RhoA/ROCK in neurons increased significantly after SCI but decreased after TT. Ultimately, we performed triple staining for NeuN/NGR/CSPG to observe the binding of CSPG produced by astrocytes to NGR on the membranes of neurons (Figure 7(b)). CSPG binds to NGR on the membranes of neurons after injury, thus activating downstream signaling pathways. The number of colabeled cells was lower in the TM group than in the M group.

## 4. Discussion

**Figure 8:**
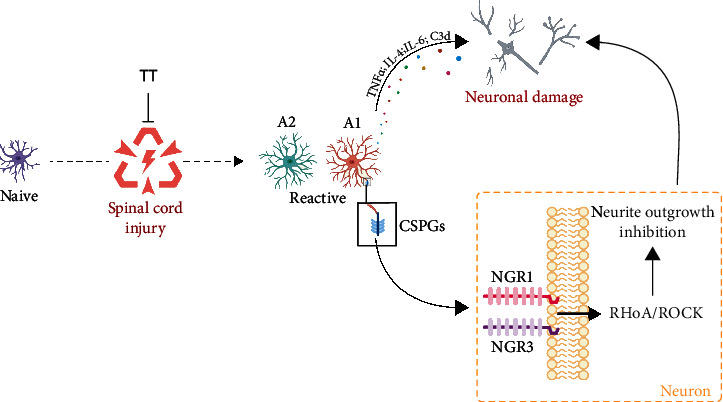
Proposed mechanism by which TT protects the neurons after SCI. After SCI, “Naive” astrocytes are activated, and “Reactive” astrocytes secrete inflammatory mediators to directly damage neurons. In addition, CSPGs will bind to NGR1, 3, activate downstream RhoA/ROCK signaling pathway to inhibit neurite outgrowth, which indirectly damage neurons. TT can effectively inhibit the activation of astrocytes to promote axonal outgrowth after SCI.

General drug intervention barely promotes effective growth in retracted axons that is needed to form functional connections, which has always been a challenge in SCI treatment. Rehabilitation training, as a noninvasive and effective treatment, has been gradually accepted and widely used in the clinic, but the specific mechanism underlying its effect on SCI has not been fully elucidated. In this study, we found that water treadmill training played a novel role in recovery from SCI by promoting axonal outgrowth associated with the NGR/RhoA/ROCK signaling pathway by inhibiting astrocyte activation ((Figure 8).

Early rehabilitation exercise after SCI can maintain muscle strength and prevent joint contracture [24, 25]. Previous studies have shown that the expression of neurotrophin increases after a threshold of TT intensity in rats with SCI [17, 26]. Furthermore, TT can also attenuate disruption to the blood–spinal cord barrier [22], which provides nutrition for nerve cells. Focusing on the paralysis of lower limbs after spinal cord injury, we combined swimming with a treadmill to allow rats to perform active rehabilitation training through the synergistic effect of water flow and the conveyor belt. The current experimental results show that the motor function of rats was significantly improved after treadmill training for 14 d. In addition, GAP43 and NF200 were used to evaluate neuronal and axonal injury [27–29]. As expected, we found that the expression of GAP43 and NF200 significantly decreased due to neuronal injury. However, we were surprised to find that after 14 d of training, the expression of GAP43 and NF200 in the penumbra increased significantly, accompanied by an increase in the number of regenerated neurons, which indicates the neuroprotective effect of TT. To further explore the underlying mechanism of TT, we first focused on the signaling molecules in neurons.

NgR1 and NgR3, members of the Nogo receptor family, bind to CSPG, activating a signaling cascade via Rho family GTPases [9, 30, 31]. RhoA is the most important Rho family gene associated with nerve regeneration [7]. The main signaling molecule downstream of RhoA is Ras homolog associated kinase [32]. RhoA/ROCK activation, in turn, activates downstream effectors, which regulate cytoskeletal reorganization such as growth cone collapse and neurite outgrowth inhibition [33]. Studies have shown that inhibiting RhoA/ROCK improves neurite outgrowth [14, 15]. Furthermore, inhibiting ROCK with Y-27632 promotes axonal regeneration after SCI [11, 16]. Interestingly, we found that activation the RhoA/ROCK signaling pathway in neurons increased significantly after SCI but decreased significantly after TT (Figure 6). As expected, the experimental results suggest that the neuroprotective effect of TT after SCI is closely associated with the RhoA/ROCK signaling pathway. It is worth pointing out that the specific inhibitor of ROCK (Y27632) has no effect on the neurological function of normal rats, which excludes the effect of the inhibitor itself on the experiment (Figure 1). The simple use of Y27632 inhibits the RhoA/ROCK signaling pathway and improves neurological function after SCI in rats. However, the effect of TT intervention was even larger (Figure 6), which indicates that TT can be used as a nondrug means to reduce the inhibition of axonal growth in SCI rats and thus promote the recovery of nerve function. Therefore, we conducted additional investigations.

Glial cells, which are the most abundant cell type in the CNS, play vital roles in maintaining CNS homeostasis [5]. Astrocytes exhibit morphological and functional differences after nociceptive stimulation, including (1) morphological changes, (2) proliferation, (3) changes in gene expression, (4) significant molecular changes, and (5) functional changes [6]. Immediately after SCI, oxidative stress causes microglial and astrocyte activation; these cells release excessive amounts of proinflammatory mediators, such as IL-1*β*, IL-6, and TNF-*α*, which are directly deleterious to neighboring neurons [34, 35]. As expected, in our experiment, we found that the abundant expression of proinflammatory factors (IL-1*β*, IL-6, and TNF-*α*) induced by SCI decreased significantly after TT (Figure 4). The fluorescence staining results showed a significantly higher number of hypertrophic astrocytes following SCI, and the Ki67/GFAP costaining results showed that many astrocytes proliferated after SCI. Furthermore, our study also suggests that injury induces the activation of microglia. By comparing the results of GFAP/Iba1 costaining between the S group and M group, we found that these two cell types were located in close proximity after activation, indicating that there may be coactivation between these cells. In contrast, this phenomenon was alleviated in the TM group, indicating that TT can significantly inhibit the activation of astrocytes and microglia.

After noxious stimulation and nerve injury, the phenotype, functions, and gene expression of astrocytes undergo significant changes known as reactive astrogliosis [5]. Consistent with the results of the latest stroke study, we found that SCI induced the activation of two kinds of astrocytes: neurotoxic A1 astrocytes, which release the neurotoxic complement protein C3d, resulting in neuronal death, and neuroprotective A2 astrocytes, which release S100A10 to protect neurons [4]. The dual roles of reactive astrocytes after SCI have prompted further research. For example, is it possible that targeting the transformation of A1- and A2-reactive astrocytes is a strategy for the prevention and treatment of SCI? As we hypothesized, the expression of the A1- and A2-type marker proteins C3d and S100A10, respectively, was significantly upregulated around the lesion after SCI. However, after TT, the number of A1 RAs decreased significantly, while the number of A2 RAs increased, suggesting that TT can inhibit A1-type RAs and promote A2-type RAs (Figure 5).

Subsequently, RAs proliferate, migrate, and transform into SAs [2, 3, 36]. The most representative marker gene of SAs is CSPG, which binds to NGR1 and NGR3 on neurons and activates the downstream RhoA/ROCK signaling pathway to inhibit neurite growth [6, 37]. In light of previous studies, we examined the expression of CSPG and its localization and binding in neurons after SCI. We found that 7 d after injury, a large amount of CSPG was secreted by astrocytes around the injury site, but the expression level decreased significantly in the TT group. The expression of CSPG was downregulated 14 d after injury, especially in the TM group (Figure 6). In addition, the number of NeuN^+^/NGR^+^/CSPG^+^ cells in the M group was significantly higher than that in the S group, indicating that a large amount of CSPG in the extracellular matrix bound to NGR on neurons after injury. We were surprised to find that the number of colabeled cells in the TM group was much lower than that in the M group (Figure 7). The therapeutic effect of TT may be associated with a decrease in the number of activated SAs, which would also explain the root cause of the decrease in RhoA/ROCK activation in the neurons in the TM group. In summary, these results suggest that TT can effectively promote axonal growth after SCI, partly by regulating RA activation to reduce activation of the RhoA/ROCK signaling pathway in neurons.

However, it is important to note the major limitation of this study: we only included male rats, and our results therefore ignore any possible differences in TT treatment caused by sex. To our knowledge, there is currently no effective treatment for SCI that can promote the growth of injured axons without any side effects. Fortunately, our team has been working on developing a motor therapy that includes early treatment intervention to promote axonal growth. The aim is to translate this method into a clinical treatment upon elucidation of the underlying mechanism.

## 5. Conclusion

In conclusion, our study presented proof that TT ameliorates neurite outgrowth inhibition. Additionally, TT decreased the activation of astrocytes after SCI, decreased the expression of A1 astrocytes, and increased the expression of A2 astrocytes. Ultimately, TT reduced the CSPGs, secreted by SAs, thus reducing the activation of RhoA/ROCK signaling pathway, which indirectly reduced the inhibition of neurite outgrowth (Figure 8).

## Data Availability

The data used to support the findings of this study are included within the article and available from the corresponding author on reasonable request.

## Abbreviations

SCI: Spinal cord injury
RAs: Reactive astrocytes
SAs: Scar-formed astrocytes
CSPGs: Chondroitin sulfate proteoglycan
NGR: Nogo receptor
h: Hour
d: Day
CNS: Central nervous system
Brdu: 5-Bromo-20-deoxyuridine
GFAP: Glial fibrillary acidic protein
Iba1: Ionized calcium binding adapter molecule 1
NF200: Neurofilament-200
RhoA: Ras homolog gene family, member A
IL-1*β*: Interleukin-1*β*
IL-6: Interleukin-6
TNF*α*: Tumor necrosis factor *α*
GAP43: Growth associated protein-43
S100A10: S100 calcium-binding protein A10
CSPGs: Chondroitin sulfate proteoglycans
NeuN: Neuron-specific nuclear protein
TT: Treadmill training
BBB: Basso-Beattie-Bresnahan
HE: Hematoxylin-eosin
BCA: Bicinchoninic acid
TBS: Tris-buffered saline solution
DAPI: 4,6-Diamidino-2-phenylindole.
